# Peripheral blood mononuclear cells exhibit increased mitochondrial respiration after adjuvant chemo‐ and radiotherapy for early breast cancer

**DOI:** 10.1002/cam4.6333

**Published:** 2023-07-13

**Authors:** Ida Bager Christensen, Marie‐Louise Abrahamsen, Lucas Ribas, Kristian Buch‐Larsen, Djordje Marina, Michael Andersson, Steen Larsen, Peter Schwarz, Flemming Dela, Linn Gillberg

**Affiliations:** ^1^ Xlab, Department of Biomedical Sciences University of Copenhagen Copenhagen Denmark; ^2^ Department of Endocrinology Rigshospitalet Copenhagen Denmark; ^3^ Department of Oncology Rigshospitalet Copenhagen Denmark; ^4^ Clinical Research Centre Medical University of Bialystok Bialystok Poland; ^5^ Faculty of Health and Medical Sciences University of Copenhagen Copenhagen Denmark; ^6^ Department of Geriatrics Bispebjerg University Hospital Copenhagen Denmark

**Keywords:** breast cancer, chemotherapy, energy metabolism, high‐resolution respirometry, mitochondria, peripheral blood mononuclear cells

## Abstract

**Background:**

Adjuvant chemo‐ and radiotherapy cause cellular damage to tumorous and healthy dividing cells. Chemotherapy has been shown to cause mitochondrial respiratory dysfunction in non‐tumorous tissues, but the effects on human peripheral blood mononuclear cells (PBMCs) remain unknown.

**Aim:**

We aimed to investigate mitochondrial respiration of PBMCs before and after adjuvant chemo‐ and radiotherapy in postmenopausal patients with early breast cancer (EBC) and relate these to metabolic parameters of the patients.

**Methods:**

Twenty‐three postmenopausal women diagnosed with EBC were examined before and shortly after chemotherapy with (*n* = 18) or without (*n* = 5) radiotherapy. Respiration (O_2_ flux per million PBMCs) was assessed by high‐resolution respirometry of intact and permeabilized PBMCs. Clinical metabolic characteristics and mitochondrial DNA (mtDNA) content of PBMCs (mtDN relative to nuclear DNA) were furthermore assessed.

**Results:**

Respiration of intact and permeabilized PBMCs from EBC patients significantly increased with adjuvant chemo‐ and radiotherapy (*p* = 6 × 10^−5^ and *p* = 1 × 10^−7^, respectively). The oxygen flux attributed to specific mitochondrial complexes and respiratory states increased by 17–43% compared to before therapy initiation. Similarly, PBMC mtDNA content increased by 40% (*p* = 0.002). Leukocytes (*p* = 0.0001), hemoglobin (*p* = 0.0003), and HDL cholesterol (*p* = 0.003) concentrations decreased whereas triglyceride (*p* = 0.01) and LDL (*p* = 0.02) concentrations increased after treatment suggesting a worsened metabolic state. None of the metabolic parameters or the mtDNA content of PBMCs correlated significantly with PBMC respiration.

**Conclusion:**

This study shows that mitochondrial respiration and mtDNA content in circulating PBMCs increase after adjuvant chemo‐ and radiotherapy in postmenopausal patients with EBC. Besides the increased mtDNA content, a shift in PBMC subpopulation proportions towards cells relying on oxidative phosphorylation, who may be less sensitive to chemotherapy, might influence the increased mitochondrial respiration observed iafter chemotherapy.

## 1 INTRODUCTION

Breast cancer was the most frequently diagnosed malignancy in 2020, with 2.3 million new cases globally.^1^ Fortunately, breast cancer patients have one of the highest five‐year relative survival rates of 90%,^2^ attributed to earlier detection due to mammography screening programs and improved treatment options.^3^, ^4^ Early (non‐metastatic) breast cancer (EBC) therapy is often multimodal, combining surgery and adjuvant treatments including chemotherapy, radiotherapy, and/or anti‐estrogen treatment combined to minimize cancer recurrence risk, drug resistance, and undesired side effects.^3^, ^5^ However, these treatment regimens are associated with whole‐body metabolic derangements and endocrine side effects.^6^, ^7^, ^8^, ^9^, ^10^ Among the documented metabolic side effects are weight gain,^11^, ^12^ increased fasting insulin and HbA1c^13^ alongside insulin resistance,^14^ increased risk of type 2 diabetes,^15^, ^16^ and cardiovascular disease.^17^, ^18^, ^19^ Changes in lipid profile, for example decreased HDL cholesterol and increased triglyceride concentrations are also described after chemotherapy.^9^, ^14^ Thus, after chemotherapy, metabolic deterioration in breast cancer patients is well established but underlying mechanisms have not yet been fully elucidated.

Cytotoxicity of chemotherapeutic drugs to malignant cells is achieved by interference with growth kinetics, for example, via direct DNA alkylation damage,^20^ intercalation of drug molecules into nucleic acids,^21^ microtubule disruption,^22^ or enzymatic interference.^23^ Radiotherapy deposits high‐energy ionizing radiation localized onto malignant tissues inducing DNA damage and cell death.^24^ However, chemotherapy and local radiotherapy are not restricted to target cancer cells but affect DNA and cellular functions in healthy cells as well.^25^, ^26^ For instance, chemotherapy has been shown to induce mitochondrial dysfunction in skeletal and cardiac muscle tissue.^27^, ^28^, ^29^, ^30^, ^31^ Repeated administration of the chemotherapeutic anthracycline doxorubicin in mice impairs mitochondrial respiration in skeletal myocytes.^27^ Moreover, chemotherapy has been shown to cause deletion and oxidation of mitochondrial DNA and reduced activity of electron transport chain complexes in murine cardiomyocytes.^28^, ^29^, ^30^, ^31^ The effect of different antineoplastic drugs on mitochondrial oxygen consumption has been investigated by incubation of cells in vitro.^32^, ^33^ In one study, the alkylating agent cisplatin caused no distinct impairment of oxygen consumption. In contrast, the anthracyclines doxorubicin and daunorubicin inhibit mitochondrial respiration in leukemia and lymphoma cells.^32^ Radiotherapy is known to damage nuclear DNA but a direct effect on complex I, II, and III in the electron transport chain and ATP synthase activity has also been described, suggesting that radiation can affect oxidative phosphorylation (OXPHOS).^34^ Interestingly, a recent study on skeletal myocytes from patients with EBC reports unchanged mitochondrial respiratory capacity but significantly reduced mitochondrial biogenesis and exacerbated H_2_O_2_ production after adjuvant chemotherapy.^35^ To shortly summarize, antineoplastic agents have been shown to affect mitochondrial function including respiratory capacity in various cell types exceeding cancer cells.

Mitochondrial respiration of peripheral blood mononuclear cells (PBMCs) has attracted attention since an increasing number of studies indicate respiratory impairment of PBMCs in several disease states, including septic shock,^36^ end‐stage renal disease,^37^ age‐related fatigue,^38^ chronic fatigue syndrome,^39^ and major depression disorders.^40^ PBMCs are a heterogenous population of cells who support immune function. Knowledge of how systemic chemotherapy and localized radiotherapy affect immune cells is of great importance. We hypothesized that systemic chemotherapy negatively affects the respiratory function of PBMCs. Thus, we aimed to investigate how mitochondrial respiration of PBMCs is affected by adjuvant chemotherapy administered with or without radiotherapy to postmenopausal EBC patients.

## 2 MATERIALS AND METHODS

### 2.1 Clinical study

The clinical study has been described previously.^14^ Briefly, postmenopausal EBC patients eligible for adjuvant chemotherapy were recruited to the clinical trial named *Healthy Living after Breast Cancer* (NCT03784651) at the Department of Oncology, Rigshospitalet, Copenhagen, Denmark. All postmenopausal patients included were aged 50–70 years, diagnosed with stage I‐III EBC, and had not yet started chemotherapy or local radiotherapy at the time of recruitment. Exclusion criteria were preexisting endocrine or metabolic disease (e.g., type 2 diabetes, osteoporosis, or thyroid disease) and malignancy before the current breast cancer diagnosis. Patients included in the clinical trial are examined every 6 months in the first 2 years and yearly for 5 years in this observational, longitudinal follow‐up study. Data presented in this article include patients with 23 examined before adjuvant chemotherapy (pre‐adjuvant therapy) and after completion of chemotherapy with (*n* = 18) or without (*n* = 5) radiotherapy (post‐adjuvant therapy). Most patients received a combination of paclitaxel, cyclophosphamide, and epirubicin as adjuvant chemotherapy (78%), with an average time between the first and last treatment of 112 days (ranging from 85 to 163 days). Seven patients received neoadjuvant (e.g., before surgery) chemotherapy and 18 patients received local radiotherapy. For the 16 patients treated with adjuvant chemotherapy after tumor resection, pre‐adjuvant therapy blood sampling was performed on average 36 days (range 27–61 days) after surgery. Post‐adjuvant therapy blood sampling was taken on average 81 days (range 29–178 days) after completion of chemotherapy.

In this paper, adjuvant therapy refers to all patients receiving chemotherapy with or without radiotherapy. Patients with estrogen receptor‐positive disease had initiated endocrine therapy (aromatase inhibitors) when post‐adjuvant therapy blood sampling was performed (*n* = 20). Anthropometric measurements (e.g., height, weight, and body mass index) and blood sampling of the patients were performed at the Department of Endocrinology and Metabolism, Rigshospitalet.^14^ The patients' tumor characteristics, surgery, and chemotherapy regimens (Table S1) were documented at the Department of Oncology, Rigshospitalet and analysis of blood samples (fasting glucose, fasting insulin, HbA1c, total cholesterol, HDL cholesterol, LDL cholesterol, triglyceride, C peptide, hemoglobin, leukocytes) were performed at the Department of Clinical Biochemistry, Rigshospitalet. Metabolic characteristics on 38 patients included in the *Healthy Living after Breast Cancer* study are published.^14^ Here, we present data on 23 patients, nine of whom were included in the publication by Buch‐Larsen et al.^14^

### 2.2 Peripheral blood mononuclear cell isolation

Venous blood (9 or 15 mL) was sampled in K_2_EDTA tubes, diluted in phosphate buffered saline (PBS; Gibco) to a final volume of 30 mL, carefully layered on top of 15 mL Lymphoprep™ density gradient medium (StemCell Technologies Inc.), and centrifuged at 800 *g* for 15 min (acceleration 9, deceleration 2) to separate PBMCs from granulocytes and erythrocytes. PBMCs were gently collected with a Pasteur pipette and transferred to a separate tube. Isolated PBMCs were washed with PBS and centrifuged at 433 *g* for 5 min (acceleration 9, deceleration 9). The supernatant was discarded, and the PBMC pellet was re‐suspended in 1 mL of Mitochondrial Respiration media 05 (MiR05, content described below). The amount of PBMCs was quantified by the NucleoCounter® NC‐3000 using the NucleoView NC‐3000 software (ChemoMetec).

### 2.3 Polarographic measurement of oxygen consumption

PBMC respiration was measured by high‐resolution respirometry using Oxygraph‐O2k instruments (Oroboros Instruments) at 37°C and 750 rpm stirring. Oxygen consumption over time and the derivative oxygen flux (O_2_ flux) was recorded by DatLab 6.1.0.7 software (Oroboros Instruments). Instrumental background calibration was routinely performed according to the manufacturer's guidelines. All experiments were performed in 2 mL chambers containing MiR05 (110 mM sucrose, 60 mM potassium lactobionate, 0.5 mM EGTA, 3 mM MgCl_2_ · 6H_2_O, 20 mM taurine, 10 mM KH_2_PO_4_, 20 mM HEPES, 1 g/L BSA, pH 7.1). Oxygen calibration at air saturation was performed prior to each experiment. Measurements of O_2_ flux per million PBMCs were based on the current barometric pressure, the constantly measured oxygen concentration in the chamber, and the oxygen solubility factor of MiR05 (0.92).^41^ Experiments were performed at an oxygen concentration ranging from 0 to 200 μM. Oxygen concentration and O_2_ flux were measured by polarographic oxygen sensors over time, determining the oxygen consumption rate per million PBMCs. Based on PBMC quantification results, a cell suspension volume corresponding to 1.5 million PBMCs was determined. This volume of MiR05 was removed from the Oxygraph‐O2k chambers, and the same volume of cell suspension was added.

After addition of PBMCs to the Oxygraph‐O2k chambers, oxygen consumption and flux were continuously recorded, and experiments were conducted by adding substrates and inhibitors in a predetermined order to distinguish oxygen consumption attributed to different mitochondrial complexes and respiratory states. Two protocols were applied, and measurements were performed in duplicate. The predetermined order of substrates and inhibitors inducing respiration attributed to specific mitochondrial complexes were as followed:

*Intact PBMCs*: 1. Endogenous routine respiration (no substrate addition); 2. Oligomycin addition (4 μg/mL) to estimate proton leak over the inner mitochondrial membrane including non‐mitochondrial respiration; 3. Carbonylcyanide *p*‐trifluoromethoxyphenylhydrazone (FCCP) titration (0.25 μM, titration until the decline of O_2_ flux) to estimate the maximal capacity of the electron transport system (ETS). This protocol examined the cellular respiration of intact PBMCs with endogenous substrates available. Oligomycin inhibits ATP synthase, and FCCP uncouples OXPHOS from the electron transport chain by collapsing the electrochemical gradient across the inner mitochondrial membrane.^42^

*Permeabilized PBMCs*: 1. Endogenous routine respiration (no substrate addition); 2. Digitonin addition (5 μg/mL DMSO) to permeabilize the PBMCs; 3. Simultaneous Malate (2 mM), Glutamate (10 mM), and Pyruvate addition (5 mM) to estimate leak respiration with the presence of complex I‐linked substrates (LEAK_CI_); 4. Simultaneous ADP (5 mM) and MgCl_2_ addition (3 mM) to estimate state 3 complex I‐linked respiration (CI_*P*_); 5. Cytochrome C addition (0.01 mM) to evaluate the integrity of the outer mitochondrial membrane; 6. Succinate addition (10 mM) to estimate state 3 complex I + II‐linked respiration (CI + CII_*P*_); 7. FCCP titration (0.25 μM, titration until the decline of O_2_ flux) to estimate maximal ETS capacity. This protocol examined O_2_ flux attributed to different mitochondrial complexes (CI_*P*_ and CI + CII_*P*_) and specific respiratory states (LEAK_CI_ and ETS) of PBMCs with saturating concentrations of substrates and inhibitors. Digitonin selectively permeabilizes the plasma membrane. Malate, glutamate, and pyruvate are complex I‐linked substrates. ADP is ATP synthase substrate and Mg2+ ions bind produced ATP. Cytochrome C is present in the mitochondrial intermembrane space and carries electrons from complex III to complex IV. Succinate is a complex II‐linked substrate. FCCP is described previously.

### 2.4 Mitochondrial DNA content

Mitochondrial DNA (mtDNA) content in PBMCs from the patients was estimated by measurements of mtDNA relative to nuclear DNA (ncDNA). DNA was extracted from PBMC pellets isolated on the same day as the high‐resolution respirometry was performed using the Mag‐Bind® Blood and Tissue DNA HDQ 96 Kit (Omega Bio‐Tek) and the KingFisher™ Duo Prime purification system (Thermo Scientific™). DNA concentration was measured using Qubit Fluorometric Quantification with the dsDNA Broad Range Assay Kit (Thermo Scientific™). Quantitative real‐time polymerase chain reaction (qPCR) was performed using the QuantiNova™ SYBR® Green PCR kit (Qiagen) and the QuantStudio™ real‐time cycler (Thermo Scientific™). qPCR was performed in triplicates for the mitochondrial tRNA^LEU^ gene (Forward primer: 5’‐CACCCAAGAACAGGGTTTGT‐3′, Reverse primer: 5’‐TGGCCATGGGTATGTTGTTAA‐3′) and the nuclear beta‐2‐microglobulin gene (Forward primer: 5’‐TGCTGTCTCCATG‐TTTGATGTATCT‐3′, Reverse primer: 5’‐TCTCTGCTCCCCACCTCTAAGT‐3′).^43^ Analysis of mtDNA/ncDNA ratio was performed using the delta–delta Ct method.^44^

### 2.5 Statistical analysis

Statistical analyses were performed using RStudio (v.1.3.1093). Before statistical analyses were conducted, normal distribution of data was assessed by QQ‐plots and Shapiro–Wilk tests. Moreover, variance equality was assessed by F tests and homoscedasticity plots of residuals. If assumptions for statistical tests were not met, data were log‐transformed, which is stated in figure legends. Changes in respiratory capacities of intact PBMCs, respiratory control ratios of permeabilized PBMCs and parametric clinical data after versus before adjuvant therapy were analyzed using paired Student's *t*‐tests. Wilcoxon signed‐rank tests were used for non‐parametric data, including mtDNA content. Changes in PBMC respiration following adjuvant therapy were analyzed by a linear mixed effects model with time (pre versus post‐adjuvant therapy) as a fixed effect and patient IDs as a random effect. A *p*‐value of <0.05 was considered to be statistically significant.

## 3 RESULTS

### 3.1 Changes in leukocyte count and lipid profile in EBC patients after adjuvant therapy

Anthropometric and biochemical data on the EBC patients pre‐ and post‐adjuvant therapy are shown in Table 1. The average age of the patients was 59 years. The patients had a stable weight following adjuvant therapy, and there were no significant changes in fasting glucose, insulin, C peptide, and HbA1c concentrations. When assessing the fasting lipid profile of the EBC patients before and after adjuvant therapy, we found significantly decreased HDL cholesterol, and increased LDL cholesterol and triglyceride concentrations (Table 1). Moreover, the hemoglobin concentrations and leukocyte count significantly decreased after the completion of adjuvant therapy. These results are in accordance with previously published results from this clinical study.^14^ Data on differential blood count are not available from the present study cohort.

**TABLE 1:** Characteristics of postmenopausalpatients with EBC before and after adjuvant therapy.

	Pre‐adjuvant therapy (*n* = 23)	Post‐adjuvant therapy (*n* = 23)	Paired *t*‐test *p*‐value
Age (years)	59 ± 5	N/A	N/A
Weight (kg)	75 ± 12	74 ± 12	0.43
BMI (kg/m^2^)	27 ± 4	27 ± 4	0.68
Fasting glucose (mmol/L)^a^	5.9 ± 1.0	5.8 ± 1.4	0.49
Fasting insulin (pmol/L)^a^	76 (62, 116)	89 (69, 129)	0.36
C peptide (pmol/L)^a^	780 (640, 1090)	947.0 (747, 947)	0.49
HbA1c (mmol/mol)	37.0 (35.0, 40.0)	37.0 (36.0, 39.0)	0.82
Total cholesterol (mmol/L)^a^	5.9 ± 1.1	6.1 ± 0.9	0.12
LDL cholesterol (mmol/L)^a^	3.8 ± 0.9	4.1 ± 0.8	0.02
HDL cholesterol (mmol/L)^a^	1.8 ± 0.4	1.6 ± 0.2	0.004
Triglyceride (mmol/L)^a^	1.3 ± 0.4	1.6 ± 0.8	0.01
Hemoglobin (mmol/L)^b^	8.2 ± 0.8	7.7 ± 0.5	0.0003
Leukocytes (10^9^/L)^b^	5.7 ± 1.1	4.7 ± 1.1	1 × 10^−4^

*Note*: Data are presented as mean ± SD for normally distributed variables and median (25–75% interquartile range) for variables that were not normally distributed. Statistically significant changes identified by paired Student's *t*‐test for parametric data and Wilcoxon signed‐rank test for non‐parametric data.

Abbreviations: BMI, body mass index; HbA1c, hemoglobin A1c; LDL, low‐density lipoprotein; HDL, high‐density lipoprotein; N/A, not applicable.

^a^ *n* = 21, as two patients were not assessed in a fasted state.

^b^ *n* = 22.

### 3.2 Mitochondrial respiration in PBMCs from EBC patients is increased after adjuvant therapy

**FIGURE 1:**
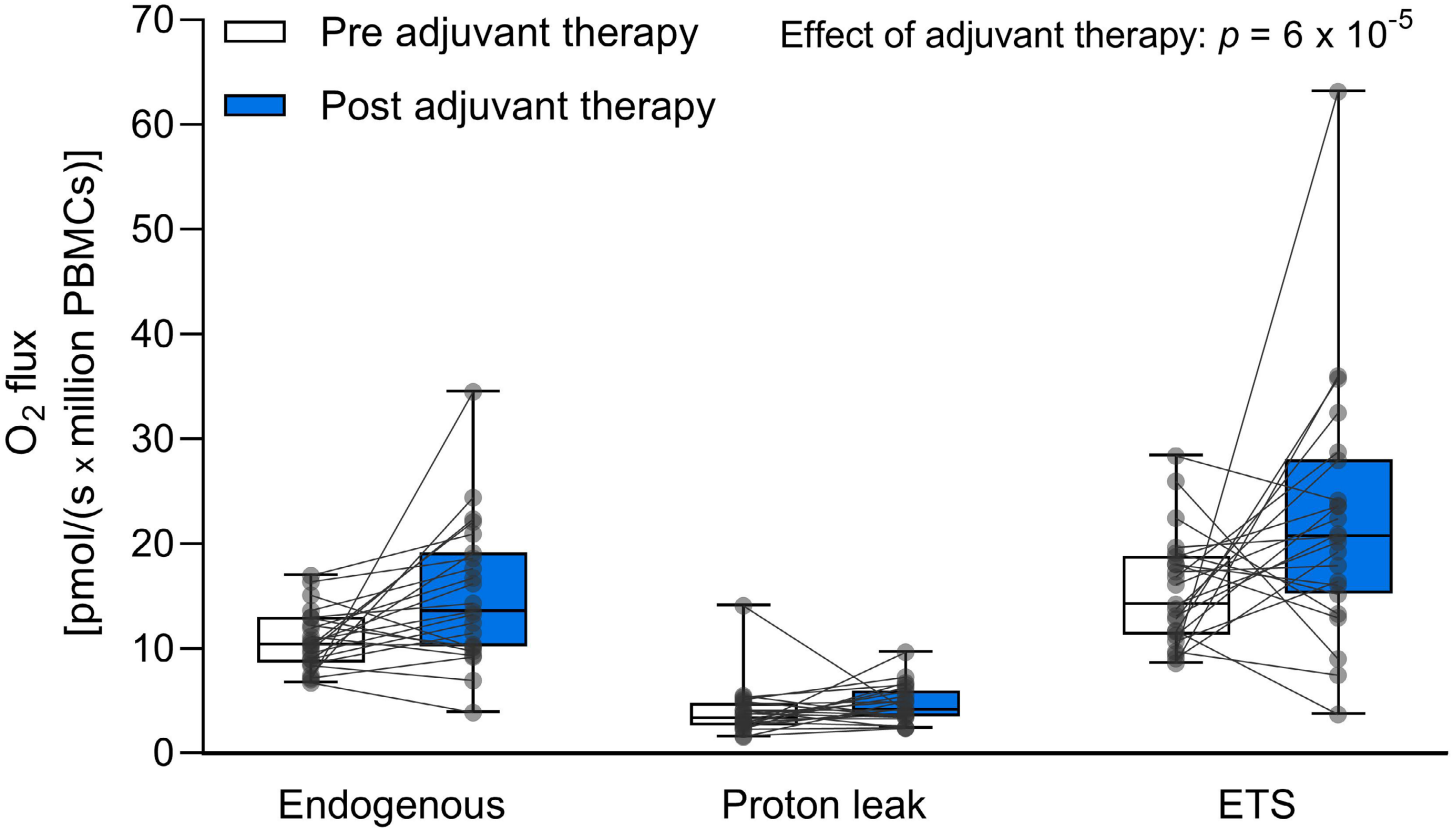
Respiration of intact PBMCs from EBC patients pre‐ and post‐adjuvant therapy. Boxplots indicate medians, interquartile range (25th–75th percentile), and minimum to maximum (whiskers) O_2_ flux. All participants are shown as individual data points. White boxes: Pre‐adjuvant therapy, *n* = 23. Blue boxes: Post‐adjuvant therapy, *n* = 23. Endogenous: Endogenous routine respiration (no substrates or inhibitors added). Proton leak: Oxygen consumed due to proton leak over the inner mitochondrial membrane including a non‐mitochondrial respiration contribution. ETS: Maximal capacity of the electron transport system (uncoupled state). Linear mixed effects model analysis identified adjuvant therapy as the main effect on the respiratory states in intact PBMCs. Data were log‐transformed prior to the statistical analysis. The p‐value takes all comparisons into account.

Noticeably, the mitochondrial respiratory capacity of various states in intact PBMCs from the EBC patients after treatment was significantly increased compared to before adjuvant therapy (*p* = 6 × 10^−5^, Figure 1). We found no significant interaction between adjuvant therapy and respiratory states, indicating no significant difference in how endogenous respiration, proton leak, and maximal respiratory capacity of the ETS responded to the course of therapy.

**FIGURE 2:**
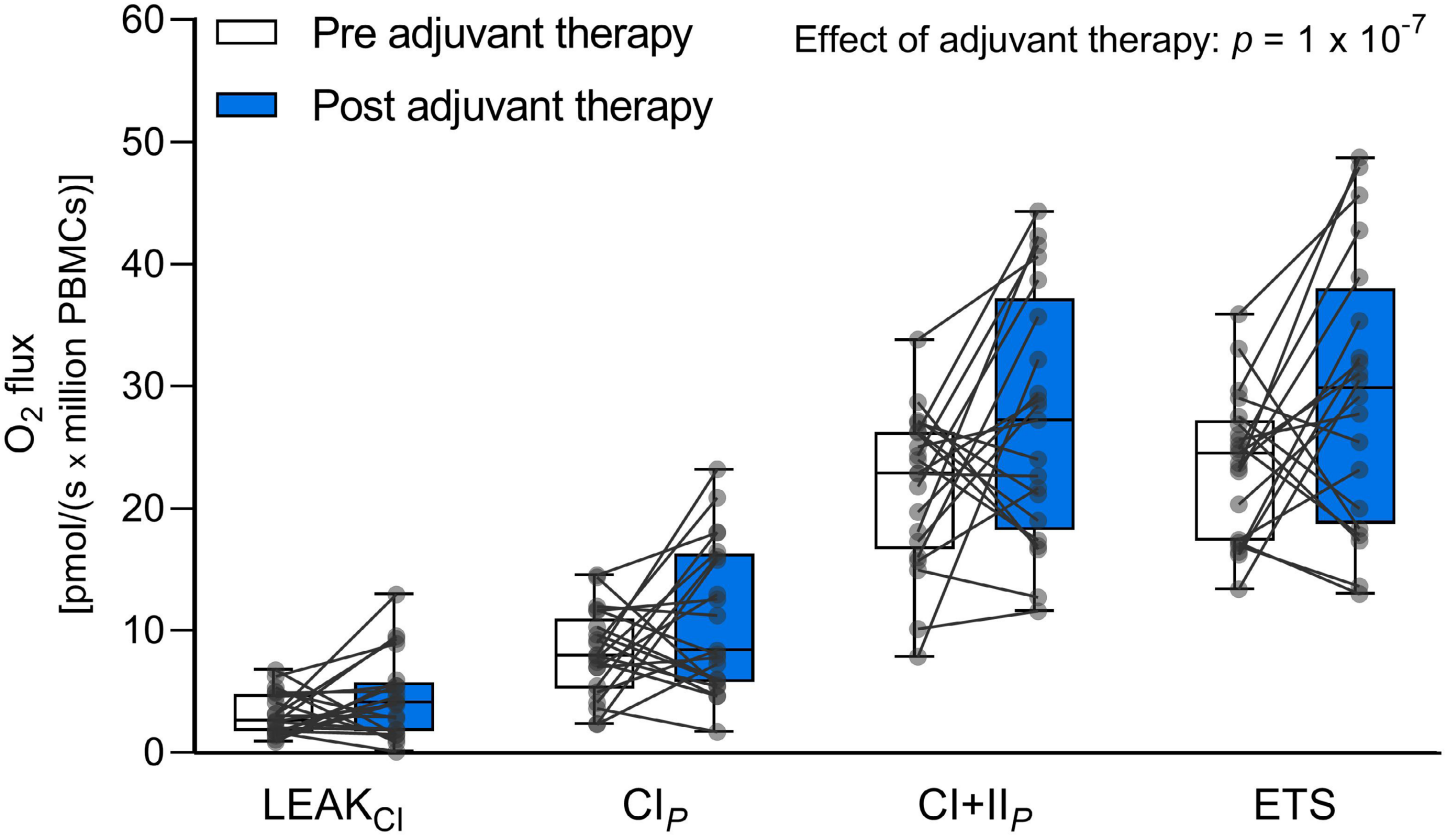
Mitochondrial respiration of permeabilized PBMCs from EBC patients pre‐ and post‐adjuvant therapy. Data are represented by boxplots indicating medians, interquartile range (25th–75th percentile), and minimum to maximum (whiskers) O_2_ flux. All participants are shown as individual data points. White boxes: Pre‐adjuvant therapy, *n* = 21. Blue boxes: Post‐adjuvant therapy, *n* = 21. LEAK_CI_: Leak respiration with the presence of complex I‐linked substrates. CI_*P*_: Complex I‐linked respiration. CI + II_*P*_: Complex I + II‐linked respiration. ETS: Maximal capacity of the electron transport system, *n* = 20. Linear mixed effects model analysis identified adjuvant therapy as the main effect on the O_2_ flux of mitochondrial complexes and respiratory states of permeabilized PBMCs. The p‐value indicated on the figure takes all comparisons into account.

To evaluate whether the increased respiration of intact PBMCs was driven by specific mitochondrial complexes or respiratory states, high‐resolution respirometry analysis of permeabilized PBMCs from the EBC patients was performed. Respiration of permeabilized PBMCs (O_2_ flux per million cells) was significantly increased after adjuvant therapy compared to before (*p* = 1 × 10^−7^, Figure 2). There was no significant interaction between adjuvant therapy and the different mitochondrial complexes and respiratory states, indicating that the observed effect of adjuvant therapy on O_2_ flux was not significantly different for specific complexes of the electron transport chain or respiratory states.

When assessing the changes in O_2_ flux for specific mitochondrial complexes and respiratory states separately, we found that the O_2_ consumption was increased for all respiratory parameters after adjuvant therapy, ranging from 17% (Proton leak) to 43% (ETS; Table 2). When evaluating the respiratory capacities of intact PBMCs and respiratory control ratios of permeabilized PBMCs (Table 3), we found the O_2_ consumption used for oxidative phosphorylation of ADP to ATP in intact PBMCs (ATP‐linked OXPHOS) to be significantly increased after adjuvant therapy versus before (*p* = 0.008).

**TABLE 2:** Percentage change in O_2_ flux from pre to post‐adjuvant therapy for specific mitochondrial complexes and respiratory states of PBMCs from EBC patients.

	Percentage change in O_2_ flux [pmol/(s × million PBMCs)]
Intact PBMCs	
Endogenous	+40%
Proton leak	+17%
ETS	+43%
Permeabilized PBMCs	
Endogenous	+38%
LEAK_CI_	+42%
CI_*P*_	+35%
CI + II_*P*_	+26%
ETS	+25%

*Note*: In the high‐resolution respirometry protocol for permeabilized PBMCs, endogenous respiration was assessed before the addition of digitonin.

Abbreviations: CI + II_*P*_, Complex I + II‐linked respiration; CI_*P*_, Complex I‐linked respiration; ETS, Maximal capacity of the electron transport system; LEAK_CI_, leak respiration with the presence of complex I‐linked substrates; PBMC, peripheral blood mononuclear cell.

**TABLE 3:** Respiratory capacities of intact PBMCs and respiratory control ratios of permeabilized PBMCs from EBC patients pre‐ and post‐adjuvant therapy.

	Pre‐adjuvant therapy	Post‐adjuvant therapy	Change (%)	Paired *t*‐test *p*‐value
Intact PBMCs				
ATP‐linked OXPHOS	6.85 ± 2.13	10.48 ± 5.35	+53%	0.008
Reserve capacity	4.73 ± 3.21	7.12 ± 6.23	+51%	0.19
Permeabilized PBMCs				
LEAK_CI_ / OXPHOS	0.15 ± 0.10	0.15 ± 0.09	0%	0.96
CI_*P*_ / CI + II_*P*_	0.37 ± 0.12	0.38 ± 0.11	+3%	0.76
OXPHOS / ETS	0.90 ± 0.12	0.94 ± 0.07	+4%	0.28
R / ETS	0.49 ± 0.08	0.53 ± 0.12	+8%	0.24

*Note*: Data are presented as mean ± SD of respiratory capacities of intact PBMCs (*n* = 23) and respiratory control ratios of permeabilized PBMCs (*n* = 21). ATP‐linked OXPHOS: Oxygen consumption used for oxidative phosphorylation (Endogenous—Leak). Reserve capacity: Respiratory reserve capacity from endogenous routine respiration to theoretical maximal capacity of the ETS (uncoupled state; ETS – Endogenous). LEAK_CI_/OXPHOS: Proportion of OXPHOS attributed to leak respiration with the presence of complex I‐linked substrates. CI_*P*_/CI + II_*P*_: Contribution of complex I to OXPHOS at fully saturated substrate concentrations. OXPHOS/ETS: Part of the maximal respiratory capacity of the ETS which OXPHOS uptakes, *n* = 20. R/ETS: Respiratory reserve capacity from routine respiration (R) to theoretical maximal capacity of the ETS, *n* = 16. Statistically significant changes were identified by paired Student's *t*‐test.

**FIGURE 3:**
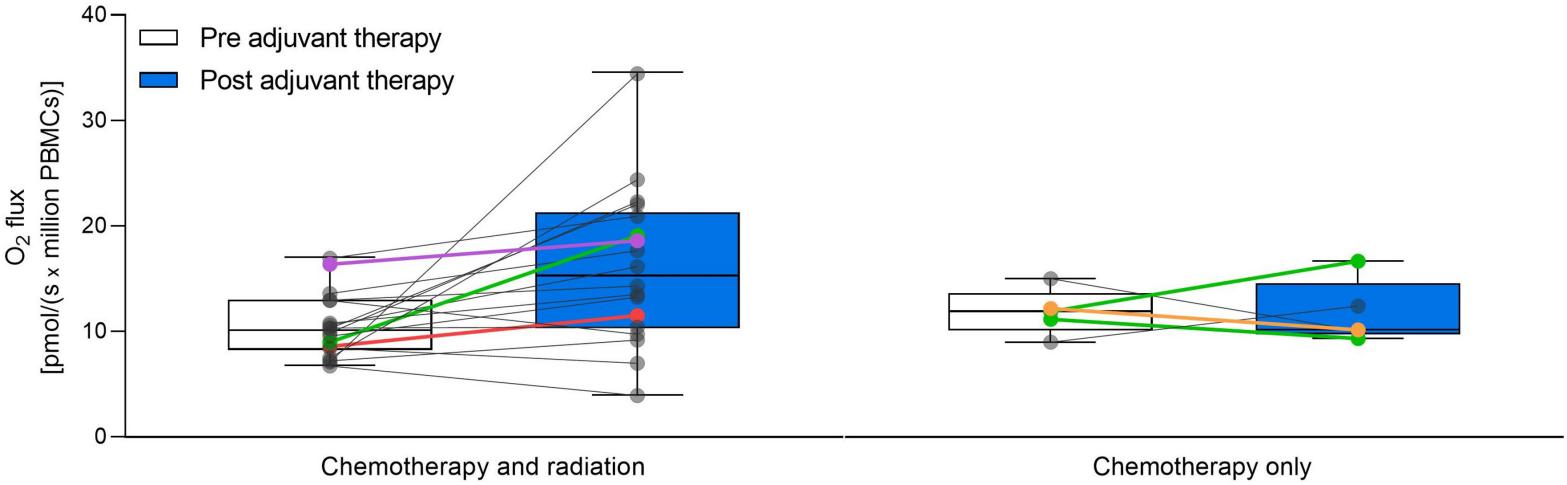
Endogenous respiration of intact PBMCs from EBC patients pre‐ and post‐adjuvant therapy. Endogenous oxygen consumption measured as O_2_ flux per million intact PBMCs from EBC patients pre‐ and post‐adjuvant therapy is presented by boxplots indicating medians, interquartile range (25th–75th percentile), and minimum to maximum (whiskers) O_2_ flux. All participants are shown as individual data points. EBC patients are stratified according to the treatment regimen, chemo‐ and radiotherapy (*n* = 18) or chemotherapy only (*n* = 5). The color of the data points indicates the chemotherapeutic drug combination used to treat the specific patient. Gray: Cyclophosphamide, epirubicin, and paclitaxel, *n* = 15 (chemo‐ and radiotherapy) and 2 (chemotherapy only). Green: Paclitaxel, *n* = 1 (chemo‐ and radiotherapy) and 2 (chemotherapy only). Red: Cyclophosphamide, epirubicin, paclitaxel, and capecitabine, *n* = 1. Purple: Cyclophosphamide and docetaxel, *n* = 1. Orange: Cyclophosphamide and epirubicin, *n* = 1. White boxes: Pre‐adjuvant therapy. Blue boxes: Post‐adjuvant therapy.

Furthermore, we investigated the impact of different treatment regimens on the endogenous respiration of intact PBMCs. EBC patients were stratified into two groups according to if they received radiotherapy (chemotherapy and radiation, *n* = 18) or not (chemotherapy only, *n* = 5). Also, EBC patients who did not receive the standard chemotherapy drug combination (cyclophosphamide, epirubicin, and paclitaxel) are marked with color. As shown in Figure 3, the significant increase in PBMC respiration after adjuvant therapy does not seem noticeably distinctive for different chemotherapeutic drug combinations. The increased endogenous respiration seems less prominent in patients receiving chemotherapy alone (*n* = 5) than patients receiving combined chemo‐ and radiotherapy (*n* = 18; Figure 3). However, this has not been statistically tested due to a relatively small sample size, rendering interpreting these effects challenging. Similarly, we lack statistical power to analyze whether endocrine therapy, which had been initiated at the post‐adjuvant therapy blood sampling for 20 (87%) of the EBC patients, affects PBMC respiration.

Because the timing of blood sampling after chemotherapy was relatively different between patients, we analyzed if the time between chemotherapy completion and post‐adjuvant therapy blood sampling had any influence on the respiratory capacities of PBMCs after chemotherapy, or the change in PBMC respirometry after vs before chemotherapy. As shown in Table S2, our data do not indicate that samples collected close to chemotherapy completion had significantly higher or lower respiration compared to samples collected several months after chemotherapy completion.

Lastly, we investigated the possible effect of breast tumor presence at the time of sampling. At the pre‐adjuvant therapy visit, patients who did not have their tumor removed (*n* = 7) did not have a different PBMC respiratory capacity from patients who had the tumor removed by surgery (*n* = 16; Table S3), indicating that the primary tumor burden did not influence the PBMC respiratory capacity in EBC patients.

### 3.3 Mitochondrial DNA content is increased in PBMCs of patients with EBC after adjuvant therapy

**FIGURE 4:**
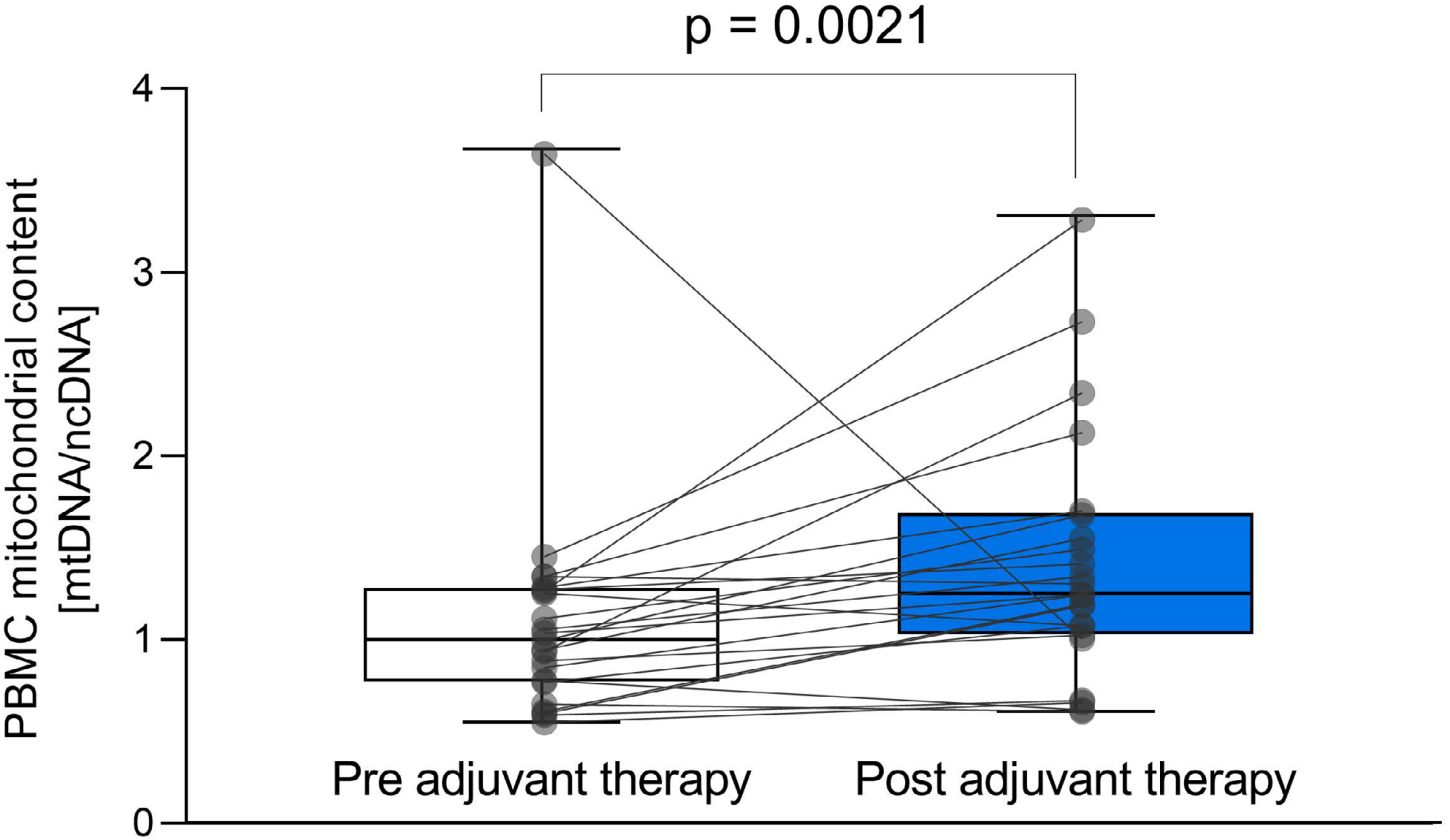
Mitochondrial DNA (mtDNA) content relative to nuclear DNA (ncDNA) in PBMCs from EBC patients before and after adjuvant therapy. Relative quantification was performed in PBMC samples from all 23 EBC patients before and after adjuvant therapy using qPCR by amplification of the mitochondrial tRNA^LEU^ gene normalized to the nuclear beta‐2‐microglobulin gene. The increase in mtDNA relative to ncDNA is indicative of increased mitochondrial DNA copy number/content. Statistical difference was calculated using Wilcoxon signed‐rank test.

We used mtDNA relative to ncDNA as a measure of mtDNA content in the patients' PBMCs and found a 40% increase after adjuvant treatment (*p* = 0.002, Figure 4). Relative mitochondrial DNA content did not correlate significantly with the O_2_ flux for endogenous respiration or any of the specific mitochondrial complexes and respiratory states of intact or permeabilized PBMCs. Also, the changes in PBMC respiration did not correlate significantly with changes in PBMC mithochondiral content. Nonetheless, intrinsic respiration (O_2_ flux corrected for mtDNA content) of intact (*p* = 0.88) and permeabilized (*p* = 0.34) PBMCs was not significantly different after compared to before adjuvant treatment.

### 3.4 No associations between PBMC respiratory capacity and metabolic characteristics

Lastly, we investigated if endogenous PBMC respiration, proton leak, or ETS capacity showed any association with age, BMI, fasting LDL and HDL cholesterol, triglycerides or glucose concentrations in the EBC patients before adjuvant treatments, which has previously been observed in healthy human subjects.^45^ We found no significant association between endogenous respiration, proton leak or maximal ETS capacity of intact PBMCs and metabolic characteristics (Table S4).

## 4 DISCUSSION

In this study, we investigated how adjuvant chemotherapy with or without radiotherapy affected mitochondrial respiration and mtDNA content in PBMCs from postmenopausal EBC patients. Interestingly, we found that respiration of intact PBMCs and respiration attributed to specific mitochondrial complexes and respiratory states of permeabilized PBMCs were significantly increased after adjuvant chemo‐ and radiotherapy. Thus, our data suggest that PBMCs in EBC patients require more oxygen to support cellular functions after therapy completion. The impact on specific mitochondrial complexes and respiratory states was not significantly different, indicating a generally increased oxygen consumption of PBMCs post‐adjuvant therapy, which was not driven by specific components of the mitochondria. However, ATP‐linked OXPHOS in intact PBMCs was significantly increased, and since proton leak was increased to a lower extent than other respiratory states, this could all together indicate a better coupling of the electron transport chain to OXPHOS in the PBMCs after adjuvant therapy. The number of patients included in this study limits the evaluation of how different treatment regimens impact PBMC respiration. However, the increase was more pronounced in patients receiving both chemo‐ and radiotherapy (78% of the patients). Because PBMC respiratory capacity was similar in EBC patients with versus without a tumor present at pre‐adjuvant therapy blood sampling, we do not expect that presence of a primary breast tumor impact PBMC respiration significantly. The timing of post‐adjuvant therapy blood sampling after chemotherapy completion did not show associations with respiratory capacity of the PBMC either. These interindividual differences underline the heterogenicity of the population of EBC patients, which are all assessed and treated as individual cases.

In a previous study, a rise in mtDNA/nDNA of PBMCs was associated with improved respiration of PBMCs 8–14 (but not 3–4) days after sepsis in children, which supports mitochondrial biogenesis as a mechanism of metabolic recovery over time.^46^ Because of the assumption that mitochondrial respiration is related to the cellular content of mitochondria, we estimated the mtDNA content based on mtDNA relative to ncDNA in PBMCs from the EBC patients. Comparable to the rise in oxygen flux, mtDNA content increased by, on average, 40% after versus before adjuvant therapy. Despite the concordant increase, we found no significant association between the mtDNA content and mitochondrial respiration in PBMCs, or the therapy‐induced changes, from the patients. This could be due to a discrepancy between mitochondrial content and mtDNA content per cell (e.g., if mtDNA damage or degradation is different from ncDNA damage) or different effects from the treatment combinations on mtDNA, which may blur the correlation between mtDNA content and PBMC respiration in the patients. Still, the rise in mtDNA content most likely contributes to the increased mitochondrial respiration in PBMCs seen after chemo‐ and radiotherapy, as there was no significant difference in PBMC respiration before versus after adjuvant therapy when respiration was normalized to mtDNA/ncDNA.

Another contributing factor to the increased PBMC respiration and mtDNA content after chemotherapy could be a shift in immune cell proportions after chemo‐ and radiotherapy. The population of PBMCs in peripheral blood consists of T cells, B cells, NK cells, monocytes, and dendritic cells in varying amounts,^47^ and utilization of OXPHOS for energy production is different in the different cell types.^48^ Resting naive T cells, antigen‐specific memory T cells, and FoxP3+ regulatory T cells rely primarily on mitochondrial OXPHOS for ATP generation, whereas proliferative T helper cells and cytotoxic effector T cells utilize glycolysis to a higher extent.^55^, ^56^, ^57^, ^58^ Studies on the effect of chemo‐ and radiotherapy on leukocytes in peripheral blood from breast cancer patients are not consistent. Some,^49^, ^50^, ^51^, ^52^, ^53^ but not all,^54^ studies report significant reductions in total lymphocytes and/or total number of T cells with a persistent decrease in T helper cells in breast cancer patients after chemo‐ and/or radiotherapy. Energy metabolic profiling of B lymphocyte subsets and NK cells is less described.^48^ Although speculative, the increase in mitochondrial respiration of PBMCs post‐adjuvant therapy found in this study could indicate a shift in the subpopulation of T cells from effector T cells to naïve T cells, memory T cells, and/or regulatory T cells with higher OXPHOS utilization. Such a shift could be caused by different sensitivity, turnover, and/or recovery of PBMC subpopulations in response to adjuvant treatments.^24^, ^49^, ^51^ Additionally, it is well known that repeated chemo‐ and radiotherapy cycles can induce damage to the hematopoietic system.^59^, ^60^ Previous research has shown that hematopoietic progenitor cells can be depleted by chemo‐ and/or radiotherapy after which hematopoiesis primarily will be restored by hematopoietic stem cells.^59^ Adjuvant therapy administered to our patients might have eliminated existing hematopoietic progenitor cells and concurrently affected the bone marrow microenvironment and the generation of new blood cells. If the proportion of newly differentiated PBMCs is increased after adjuvant therapy, OXPHOS utilization might increase due to their naïve phenotype.

PBMC subpopulations during and after adjuvant chemotherapy administration might also vary because of a generally fast leukocyte cell turnover. The relatively short lifespan of leukocytes^61^ and a high degree of PBMC turnover could explain why we see increased respiration and increased mtDNA content in PBMCs as opposed to impaired mitochondrial function which has been documented in more long‐lived tissue resident cellssuch as murine skeletal myocytes or cardiomyocytes after chemotherapy exposure.^27^, ^28^, ^30^, ^31^, ^32^ Therefore, translation of effects of adjuvant therapy previously reported for long‐lived cells to short‐lived cells such as PBMCs must be done cautiously.

Many research groups have investigated mitochondrial function in breast cancer and other tumor cells before and after chemotherapy. In many of these studies, OXPHOS increase in tumor cells that are resistant to specific chemotherapy drugs.^62^, ^63^, ^64^, ^65^ In a study of triple‐negative breast cancer cells, DNA‐damaging agents (doxorubicin, carboplatin) increased both mitochondrial content and OXPHOS, whereas taxanes (paclitaxel, docetaxel) decreased mitochondrial OXPHOS.^65^ Whether chemoresistant tumor cells and PBMCs that rely on mitochondrial OXPHOS are more resistant to specific chemotherapy agents, or whether these agents have a similar influence on the tumor and bone marrow microenvironment and thereby survival and energy metabolic preferences of their resident cells, should be further studied. In line with this, possible reversibility of therapy‐induced changes to PBMC respiratory capacity during the months and years after chemotherapy may indicate healing of the bone marrow microenvironment and less inflammation.

Finally, metabolic factors may influence the vasculature and the microenvironment of peripheral blood cells. The EBC patients included in this study exhibited decreased HDL cholesterol levels and increased triglyceride and LDL cholesterol levels after versus before adjuvant therapy, which is in accordance with the findings of other studies.^9^, ^14^ Interestingly, DeConne et al. found a negative correlation between LDL cholesterol levels and maximal respiration and spare respiratory capacity in PBMCs from healthy adults, suggesting a possible link between blood cholesterol and mitochondrial respiration in PBMCs.^45^ However, such associations were not evident from our data. The increased PBMC respiration observed after chemotherapy in our study is most likely due to a generally increased ATP demand rather than increased substrate availability in the blood.

In summary, the increased oxygen demand in PBMCs from EBC patients after adjuvant therapy is most likely attributed to the increased mtDNA content in the PBMCs. Other contributing factors might be an altered PBMC population with a higher proportion of cell types relying on OXPHOS or a direct effect of adjuvant therapy on the bone marrow leading to altered hematopoiesis. PBMC respiration may reflect immune activity, which most likely is increased after adjuvant therapy due to debris left behind by dead and dying cells throughout the body. In revealing how PBMCs are affected by adjuvant breast cancer treatment, we have now expanded the understanding of immune system functioning in the growing population of EBC patients.

## 5 CONCLUSIONS

Postmenopausal EBC patients exhibit increased mitochondrial respiration in circulating PBMCs after chemotherapy with or without radiotherapy. The increased oxygen demand of PBMCs after adjuvant chemo‐ and radiotherapy may be explained by the increased mtDNA content in PBMCs after adjuvant therapy and possibly also by a shift in PBMC subpopulation proportions towards cells favoring OXPHOS. However, these mechanisms need further investigation in larger observational studies.

## AUTHOR CONTRIBUTIONS

**Ida Bager Christensen:** Formal analysis (lead); investigation (lead); methodology (supporting); writing – original draft (lead); writing – review and editing (equal). **Marie‐Louise Abrahamsen:** Formal analysis (supporting); investigation (supporting); methodology (equal); writing – review and editing (equal). **Lucas Ribas:** Formal analysis (supporting); investigation (supporting); writing – review and editing (equal). **Kristian Buch‐Larsen:** Data curation (supporting); investigation (supporting); project administration (supporting); writing – review and editing (equal). **Djordje Marina:** Data curation (supporting); investigation (supporting); project administration (supporting); writing – review and editing (equal). **Michael Andersson:** Conceptualization (equal); data curation (supporting); writing – review and editing (equal). **Steen Larsen:** Conceptualization (supporting); methodology (equal); resources (supporting); supervision (supporting); writing – review and editing (equal). **Peter Schwarz:** Conceptualization (equal); funding acquisition (supporting); project administration (equal); resources (equal); supervision (supporting); writing – review and editing (equal). **Flemming Dela:** Conceptualization (supporting); funding acquisition (supporting); project administration (supporting); resources (equal); supervision (supporting); writing – review and editing (equal). **Linn Gillberg:** Conceptualization (supporting); data curation (supporting); formal analysis (equal); funding acquisition (lead); investigation (equal); methodology (equal); project administration (lead); resources (supporting); supervision (lead); writing – original draft (equal); writing – review and editing (equal).

## CONFLICT OF INTEREST STATEMENT

All authors declare the presented research to be conducted in the absence of any conflicts of interest.

## ETHICAL APPROVAL, PATIENT CONSENT, AND CLINICAL TRIAL REGISTRATION

The clinical study was conducted according to the Declaration of Helsinki and approved by the Ethics Committee of The Capital Region, Denmark (Project number H‐18016600 approved on the June 21, 2018, and amendment 67762). Informed consent was obtained from all patients involved in the study. The clinical trial (registration number NCT03784651) was registered on www.clinicaltrials.gov on December 24, 2018.

## Supporting information


Table S1.
Table S2.
Table S3.
Table S4.

## Data Availability

The data supporting the findings of this study are available from the corresponding author upon reasonable request.
