# Supplementary material for: Peripheral blood mononuclear cells exhibit increased mitochondrial respiration after adjuvant chemo‐ and radiotherapy for early breast cancer
Source: Cancer Med. 2023 Jul 13;12(16):16985–96. doi: 10.1002/cam4.6333 (PMC10501284; doi:10.1002/cam4.6333)
Supplement: Supplementary file 1 — Table S1. Table S2. Table S3. Table S4. [file CAM4-12-16985-s001.docx]

**Appendix**

| **Supplemental Table 1** **⏐** Tumor characteristics and treatment regimens of EBC patients. | |
| --- | --- |
| Number of patients | n = 23 |
| Tumor stage at diagnosis |  |
| I | 5 (22 %) |
| II | 14 (61 %) |
| III | 4 (17 %) |
| Histology |  |
| Invasive Ductal Carcinoma | 21 (91 %) |
| Invasive Luminal/Lobular Carcinoma | 2 (9 %) |
| Laterality |  |
| Right | 11 (48 %) |
| Left | 10 (43 %) |
| Bilateral | 2 (9 %) |
| Surgery type |  |
| Mastectomy | 7 (30 %) |
| Lumpectomy | 16 (70 %) |
| Lymph node involvement |  |
| 0 | 7 (30 %) |
| 1-3 | 16 (70 %) |
| 4+ | 0 (0 %) |
| ER status |  |
| Positive | 20 (87 %) |
| Negative | 3 (13 %) |
| HER2 status |  |
| Positive | 9 (39 %) |
| Negative | 14 (61 %) |
| PAM50 ^1^ |  |
| Luminal A | 3 (16 %) |
| Luminal B | 9 (47 %) |
| Luminal C | 3 (16 %) |
| HER2 enriched | 0 (0 %) |
| Basal like | 0 (0 %) |
| Chemotherapy |  |
| Cyclophosphamide | 20 (87 %) |
| Epirubicin | 19 (83 %) |
| Docetaxel | 1 (4 %) |
| Paclitaxel | 21 (91 %) |
| Capecitabine | 1 (4 %) |
| Radiotherapy |  |
| Yes | 18 (78 %) |
| No | 5 (22 %) |
| Number of radiation cycles | 15 (67%), 25 (28%) or 30 (5%) |
| Endocrine treatment (aromatase inhibitors) |  |
| Yes | 20 (87 %) |
| No | 3 (13 %) |
| Data are presented as number of patients and percentage of the cohort specified in parenthesis. Number of radiation cycles are presented as median and 25-75% interquartile range. ER: Estrogen receptor HER2: Human epidermal growth factor receptor 2. PAM50: Prediction analysis of microarray 50. ^1^n=19. | |

**Supplemental Table 2** **⏐** Correlations between number of days between completion of chemotherapy and 1. respiratory capacity/mtDNA amount post-adjuvant therapy, and 2. change in respiratory capacity/mtDNA amount from pre-to post-adjuvant therapy, in PBMCs from EBC patients.

| Parameter | 1. Post-adjuvant therapy | 2. Change between pre- and post-adjuvant therapy |
| --- | --- | --- |
| Intact PBMCs |  |  |
| Endogenous | \| 0.1142 (-0.3244 to 0.5123) \| \| --- \| \|  \| | 0.01928 (-0.4069 to 0.4386) |
| Proton leak | 0.3426 (-0.09395 to 0.6686) | 0.1775 (-0.2654 to 0.5584) |
| ETS | 0.04548 (-0.3848 to 0.4595) | -0.05487 (-0.4669 to 0.3768) |
| Permeabilized PBMCs |  |  |
| LEAK_CI_ | -0.1884 (-0.5826 to 0.2774) | -0.2216 (-0.6050 to 0.2452) |
| CI*_P_* | -0.1118 (-0.5284 to 0.3482) | -0.2287 (-0.6097 to 0.2381) |
| CI+II*_P_* | -0.02794 (-0.4649 to 0.4200) | -0.1644 (-0.5659 to 0.3002) |
| ETS | -0.05568 (-0.4969 to 0.4084) | -0.2053 (-0.5941 to 0.2611) |
| mtDNA content | -0.4001 (-0.7039 to 0.02744) | -0.2601 (-0.6153 to 0.1829) |

Results are presented as Spearman correlation coefficients (r) with 95% confidence intervals. Endogenous: Endogenous routine respiration (no substrates or inhibitors added). Proton leak: Oxygen consumed due to proton leak over the inner mitochondrial membrane including a non-mitochondrial respiration contribution. ETS: Maximal capacity of the electron transport system (uncoupled state). LEAK_CI_: Leak respiration with the presence of complex I-linked substrates. CI*_P_*: Complex I-linked respiration. CI+II*_P_*: Complex I+II-linked respiration. PBMC: Peripheral blood mononuclear cell. Mitochondrial DNA (mtDNA) content in PBMCs was estimated by measurements of mtDNA relative to nuclear DNA (ncDNA).

**Supplemental Table 3** **⏐** Respiratory capacities of intact and permeabilized PBMCs from EBC patients who did (n=16) or did not (n=7) have their tumor removed at their pre-adjuvant therapy visit.

|  | **Tumor removed (n=16)** | **Tumor still present (n=7)** | *p*-value |
| --- | --- | --- | --- |
| Intact PBMCs |  |  |  |
| Endogenous | 11.0 ± 2.7 | 10.4 ± 3.4 | 0.68 |
| Proton leak | 4.2 ± 2.9 | 3.5 ± 1.0 | 0.36 |
| ETS | 15.9 ± 4.7 | 14.8 ± 6.9 | 0.71 |
| Permeabilized PBMCs |  |  |  |
| LEAK_CI_ | 3.1 ± 1.8 | 3.3 ± 2.0 | 0.86 |
| CI*_P_* | 7.9 ± 3.1 | 8.8 ± 4.8 | 0.67 |
| CI+II*_P_* | 22.0 ± 5.3 | 21.0 ± 9.1 | 0.80 |
| ETS | 23.8 ± 5.2 | 23.1 ± 7.9 | 0.85 |

Data are presented as mean ± SD of respiratory capacities in PBMCs from EBC patients who had their breast tumor removed by surgery (n=16 for intact and n=15 for permeabilized PBMCs) versus patients who did not yet have their tumor removed (n=7 for intact and n=6 for permeabilized PBMCs) at their pre-adjuvant therapy visit. Endogenous: Endogenous routine respiration (no substrates or inhibitors added). Proton leak: Oxygen consumed due to proton leak over the inner mitochondrial membrane including a non-mitochondrial respiration contribution. ETS: Maximal capacity of the electron transport system (uncoupled state). LEAK_CI_: Leak respiration with the presence of complex I-linked substrates. CI*_P_*: Complex I-linked respiration. CI+II*_P_*: Complex I+II-linked respiration. PBMC: Peripheral blood mononuclear cell. Statistically significant changes were identified by unpaired Student’s *t*-test.

| **Supplemental Table 4** **⏐** Correlations between mitochondrial respiration of intact PBMC and metabolic characteristics of postmenopausal EBC patients before initiation of adjuvant therapy. | | | |
| --- | --- | --- | --- |
|  | **Endogenous routine**  (n = 23) | **Proton leak**  (n = 23) | **ETS**  (n = 23) |
| Age (years) | r=0.1883 (-0.2549 to 0.5661) | r=0.2997 (-0.1410 to 0.6413) | r=-0.02626 (-0.4442 to 0.4011) |
| BMI (kg/m^2^) | r=0.1354 (-0.3049 to 0.5281) | r=-0.1448 (-0.5350 to 0.2962) | r=0.1265 (-0.3131 to 0.5215) |
| Fasting glucose (mmol/L) | r=0.2793 (-0.1628 to 0.6281) | r=-0.03234 (-0.4491 to 0.3960) | r=0.2179 (-0.2258 to 0.5868) |
| Fasting insulin (pmol/L) | r=0.2625 (-0.1804 to 0.6169) | r=0.1097 (-0.3284 to 0.5090) | r=0.2111 (-0.2326 to 0.5820) |
| Total cholesterol (mmol/L) | r=0.03373 (-0.3948 to 0.4502) | r=-0.01045 (-0.4314 to 0.4143) | r=-0.05456 (-0.4667 to 0.3770) |
| LDL cholesterol (mmol/L) | r=-0.08803 (-0.4926 to 0.3478) | r=0.1352 (-0.3052 to 0.5279) | r=-0.1726 (-0.5550 to 0.2700) |
| HDL cholesterol (mmol/L) | r=0.3027 (-0.1379 to 0.6432) | r=0.02778 (-0.3998 to 0.4454) | r=0.2604 (-0.1826 to 0.6155) |
| Triglyceride (mmol/L) | r=0.01791 (-0.4081 to 0.4375) | r=-0.03264 (-0.4493 to 0.3957) | r=-0.04776 (-0.4613 to 0.3829) |
| Results are presented as Spearman correlation coefficients (r) with 95% confidence intervals. Endogenous: Endogenous routine respiration (no substrates or inhibitors added). Proton leak: Oxygen consumed due to proton leak over the inner mitochondrial membrane including a non-mitochondrial respiration contribution. ETS: Maximal capacity of the electron transport system (uncoupled state). BMI: Body mass index. LDL: Low density lipoprotein. HDL: High density lipoprotein. | | | |
